# Ethnic Food Consumption in Italy: The Role of Food Neophobia and Openness to Different Cultures

**DOI:** 10.3390/foods9020112

**Published:** 2020-01-21

**Authors:** Giulia Mascarello, Anna Pinto, Valentina Rizzoli, Barbara Tiozzo, Stefania Crovato, Licia Ravarotto

**Affiliations:** Health Awareness and Communication Department, Istituto Zooprofilattico Sperimentale delle Venezie, Legnaro 35020, Italy

**Keywords:** Italian consumers, food attitudes, psychological trait, sociodemographic variables, ethnic food

## Abstract

While the ethnic food market has become increasingly important in Italy, the effects of the hybridization of consumption patterns have been slowed by a consolidated culinary tradition. This study investigates the relationships among ethnic food consumption, food neophobia, and openness to different cultures with sociodemographic characteristics. A sample of 1317 Italian consumers responded to an online survey. The sociodemographic profile of the neophobic consumer appears to substantially differ from that of the consumer with an attitude of openness. Neophobic respondents are males, are older than 55 years of age, are less educated, have children, are retired, have difficulty meeting their financial responsibilities, and do not eat ethnic food. Respondents who are more open to different cultures are young adults, are highly educated, have no children, are employed, and are consumers of ethnic food. The relationship between food neophobia and openness to different cultures is confirmed to be the relationship between these variables and ethnic food consumption. The measurement of these characteristics could serve as a crucial indicator for analyzing the willingness to accept elements of novelty in an increasingly multicultural society. Additionally, consumers with the neophobic trait and who are less open to different cultures might have a less varied diet that is essential to good health.

## 1. Introduction

Italy has a consolidated culinary tradition that has its roots in regional cuisine. This tradition may serve as a stabilizing factor in the eating habits of Italians that slows the globalization of consumption patterns [1,2]. However, Italy appears to be evolving with regard to food consumption. In recent decades, significant changes have occurred in the lifestyles and dietary habits of the Italian population, including an increase in dining out [3], the widespread presence of take-out and street food, a recreational attitude towards food particularly in young consumers [4], and increasing consumer demand in terms of food variety. Food polytheism is a term used to describe the current context of food consumption in Italy. Food polytheism refers to a subjective combination of food choices and highly complex and occasionally seemingly contradictory places of purchase and consumption [3,5,6,7].

### 1.1. Increasing of Ethnic Food Presence in Italy

The hybridization of food models is favored by different factors, including the globalization of markets and the mixture of populations through migration and the development of tourist exchanges with foreign countries [8]. In recent decades, many social, political, and economic changes have fostered the arrival of significant flows of new residents into Italy who have promoted the development of economic activities in the food and catering sectors that are strongly linked to their countries of origin. The number of foreign entrepreneurs working in the catering sector increased by 43.3% from 2010 to 2014, leading to a record number of 21,176 owners of foreign enterprises (12.9% of the total number) [9]. Therefore, ethnic foods are increasingly available on the Italian food market: Since 2007, ethnic food sales have increased by 93% [10]. In addition to introducing new flavors and ingredients, ethnic food has cultural significance because it represents the food cultures and traditions of populations from other countries whose presence in Italy is increasing. For these reasons, foods from the culinary traditions of distant countries have started to have an increasingly important presence in Italy. However, their success has been delayed relative to what has been observed in other Western countries and has been less overwhelming than what has been witnessed in the Anglo-Saxon countries [11].

### 1.2. Consumption of Ethnic Food: The Role of Food Neophobia and Openness to Different Cultures

Different interrelated aspects contribute to delineate consumption decisions, particularly regarding unfamiliar foods. They can be contextual (e.g., cultural or social), related to individual cognition/perception/sensation (e.g., the fear of negative consequences of eating a particular food or finding a certain type of food disgusting), or related to individual traits [12,13]. The willingness to try non-traditional ethnic foods seems to be significantly predicted by a trait known as food neophobia [14,15], which is defined as a “reluctance to eat and/or avoidance of novel foods” [16]. Several instruments have been developed to measure food neophobia as a behavior involving the rejection of foods in a particular situation and as a personal trait related to the propensity to avoid novel foods that remains constant over time and across different contexts [12]. The Food Neophobia Scale (FNS) developed by Pliner and Hobden [16] is considered a reliable and valid instrument for measuring neophobia as a personality characteristic in adults [17].

The study of this psychological trait is important because it can affect the acceptance of new foods [18] and influence consumers’ daily food choices [19,20]. Studies on children and adults suggest that food neophobia can affect the consumption of healthy foods (i.e., fruit and vegetables) [21,22] and the willingness to try healthy alternatives (e.g., meat substitutes) [23]. Additionally, neophilic individuals consume a broader variety of food [20,24,25] and have a diet higher in nutritional quality [26] than neophobes. Thus, neophobic individuals may be more exposed to nutritional risks or suffer from specific risks related to an unvaried diet.

As many studies have highlighted, sociodemographic variables are key determinants of food choices and perceptions [27,28,29]. Food preferences and acceptance seem to be associated with food exposure [30]. Education, occupation, and income influence the chances of exposure because they can favor a habit of dining out and knowledge of different cultures and gastronomic traditions. The association between these variables and food neophobia has been observed in several studies [31,32]. The influence of age [33,34,35,36,37,38] and place of residence [27] has also been observed. Therefore, individuals who are more exposed to different cultures should be less neophobic. The relationship between food neophobia and general neophobia was assessed by Pliner and Hobden [16]. Openness to different cultures seems to influence the acceptance of and willingness to try new foods. Studying this attitude in relation to food neophobia could contribute interesting information in the development of a general approach that could influence the propensity to try foods from other countries. Several survey items have been proposed by Verbeke and Poquiviqui López [39] to study the attitudes of Belgians towards Latin American ethnic foods in an approach based on the dimensions of ethnic identity defined by Laroche et al. [40].

Few studies have focused on these issues in reference to the Italian context. A number of studies have focused on Italians’ attitudes towards Eastern European foods [15], on the relation between neophobia and odor identification [41] and food preferences [42,43,44], and on the role of neophobia in determining food preferences [13]. A measure of neophobia designed for Italian primary school children has also been validated [45]. Focusing on aspects that have been shown to influence food choices is particularly interesting in a context like the Italian one, which is characterized by a deep-rooted food culture and at the same time by increasingly multicultural conditions and consequently by food polytheism.

### 1.3. Aims

In light of the above specificities of the Italian context and of the impacts of neophobia on food preferences, this study has two objectives:

- To analyze the relationships between ethnic food consumption, the psychological trait of food neophobia, and openness to different cultures in Italy; and
- To identify the sociodemographic characteristics of food neophobic consumers and of consumers who are open to different cultures.

## 2. Materials and Methods

### 2.1. Data Collection

Data were collected through a national survey conducted via the computer-assisted web interviewing method (CAWI) [46] between 8 September and 25 September 2014 by the Demetra opinioni.net firm. The sample was selected using a method that considered the stratification of the Italian population by gender and geographical area as of 31 December 2013 as determined by the Italian National Institute of Statistics (ISTAT). A sample of 1317 Italian consumers responded to a questionnaire sent to 2871 consumers for a response rate of 45.8%. The complete questionnaire includes 45 questions.

A set of questions was designed to investigate ethnic food consumption habits and risk perceptions. An overview of the consumption of ethnic food in Italy and an analysis of perceptions of risk associated with this type of food are provided in a previous article [47]. Ethnic food/products were defined as “food coming from a foreign country with gastronomic and cultural traditions different from Italian ones” [48]. In this article, several aspects are reported to frame ethnic food consumption in Italy: Whether respondents have ever eaten ethnic food in Italy (response options: Yes and no) and the ways in which respondents come into contact with ethnic cuisine (response options: Through relatives and friends, through travel, by myself, through the mass media, and other).

Food neophobia was measured with 10 items [16], and openness to different cultures was measured with 7 items [39], with response options expressed on a 1–10 Likert scale of “strongly disagree” to “strongly agree” (see Table 1 and Table 3 for response options). The original FNS was administered with a 7-point response scale. In this study, a 10-point scale was used to ensure consistency between the two measurements and the other rating scales of the questionnaire (different scales were also used by Demattè et al. [41] and Meiselman et al. [31]). Ritchey et al. [49] demonstrated through his study that excluding 2 or 4 items from the FNS improves the instrument when applied in several countries. However, we decided to maintain the original 10 items since we applied the FNS to the Italian context alone.

Personal information investigated through the questionnaire included the following: Gender, age, educational qualifications, the presence of children, occupation, location, size of the city of residence, and how well the interviewee’s income meets his or her financial responsibilities (see Table 2 and Table 4 for response options).

### 2.2. Data Analysis

Descriptive statistics were applied to all variables (the sociodemographic information listed above and whether ethnic food was consumed or not) to develop a general understanding of ethnic food consumption in the Italian context. The two scales were studied over two steps. Cronbach’s alpha coefficient was used to assess the internal consistency of the scales while a principal component analysis (PCA) (the maximum likelihood method with varimax rotation) was used to investigate the factor structure. The skewness and kurtosis of the two scales’ distributions were also analyzed. Two indices were constructed to measure the levels of food neophobia and openness to different cultures. The two indices were calculated by summing scores allocated to the 10 statements from the FNS (range 10–100) and to the 7 statements from the openness to different cultures scale (ODCS) (range 7–70). To calculate the FNS index, the positive items were reversed [16]. An analysis of variance (ANOVA) facilitated the study of differences in the levels of food neophobia and in the openness to different cultures on the basis of social information. Based on the FNS index, the respondents were divided into three groups: Neophilic (low scores), neutral (medium scores), and neophobic (high scores). One standard deviation from the mean was used as the cut-off point. This classification has been used in previous studies and is considered an effective method [14,15,32,50]. The subjects were also divided using the same method in relation to the ODCS index. Three groups were identified: Those less (low scores), moderately (medium scores), and highly (high scores) open to different cultures. The relation between the two indices was studied by means of the Pearson correlation index. Different levels of food neophobia in relation to attitudes towards different cultures were analyzed by cross tabulation. A Chi-square test was used to verify the dependence relationship between the two variables identifying the FNS and ODCS groups. Data were processed using the SPSS (Statistical Package for Social Science) software (version 20.0) for Windows (SPSS Inc. Chicago, Illinois). The level of statistical significance was set at 5% (α = 0.05).

## 3. Results

### 3.1. Characteristics of the Sample

The sample was 52.2% female and 47.8% male (in the Italian population, 51.5% were female and 48.5% male). Regarding regions of residence, the majority of respondents (35.2%) lived in southern Italy or in Sicily or Sardinia followed by those living in northwestern Italy (26.3%), those living in central Italy (19.4%), and those living in northeastern Italy (19.1%) (in the Italian population, 34.5% lived in southern Italy or in Sicily or Sardinia, 26.5% in northwestern Italy, 19.8% in central Italy, and 19.2% in northeastern Italy). In total, 22.5% of the respondents were between 18 and 34 years of age, 37% were between 35 and 54 years of age, and 40.5% were more than 55 years of age (in the Italian population over 18 years old, people in the three age classes were respectively 22.3%, 37.2%, and 40.8%). Of the respondents, 84.7% declared that they had eaten ethnic food in Italy. The majority of those who had consumed this food in Italy had come into contact with ethnic cuisine through relatives and friends (50.4%) while nearly one in four had been exposed to ethnic cuisine through their travels (24.5%), approximately one in five had been exposed to this cuisine by themselves (22.3%), and others had been exposed through the mass media (2.3%) or through other means (0.5%).

### 3.2. Food Neophobia

The internal consistency of the FNS was assessed from Cronbach’s alpha (α = 0.789). The factor structure was evaluated by means of a PCA. The scale items loaded primarily on two components, explaining 38% and 23.3% of the variance (Table 1). The first component loaded the regular items and is related to the levels of interest in trying new and ethnic foods while the second component loaded the reversed items and seems to be related to concerns regarding trying new foods. The mean FNS score of the Italian sample analyzed here was recorded as 51.1 (SD = 14.7, range 10–100). The FNS distribution had a skewness value of 0.08 and a kurtosis value of 0.31.

The investigation of levels of food neophobia observed in relation to the consumption of ethnic food and sociodemographic variables and the results of the analysis of variance conducted are presented in Table 2. The F-test results are statistically significant with regard to the following variables: Gender (*p* = 0.040), age (*p* = 0.000), educational qualifications (*p* = 0.000), the presence of children (*p* = 0.006), occupation (*p* = 0.000), meeting financial responsibilities (*p* = 0.000), and ethnic food consumption (*p* = 0.000). Levels of food neophobia were found to be higher in those who do not eat ethnic food, in males, in those older than 55 years of age, in those with no more than an elementary/lower secondary school education, in those who have children, in retirees, and in those who are experiencing many challenges in meeting their financial needs.

### 3.3. Openness to Different Cultures

The internal consistency of the ODCS was assessed by Cronbach’s alpha (α = 0.883). A PCA was used to evaluate the factor structure. The first and second principal components were found to explain 60% and 16% of the variance, respectively (Table 3). The first factor pertains to levels of comfort with social interactions with non-Italians while the second factor refers to language and mass media use. The mean ODCS value was recorded as 39.6 (SD = 13.9, range 7–70). The ODCS distribution generated a skewness value of 0.00 and a kurtosis value of −0.33.

The results of the analysis of variance conducted are reported in Table 4. The F-test produced statistically significant results for the following variables: Age (*p* = 0.000), city size (*p* = 0.002), location (*p* = 0.000), educational qualifications (*p* = 0.000), the presence of children (*p* = 0.034), occupation (*p* = 0.000), and ethnic food consumption (*p* = 0.000). More openness to different cultures was observed in those who eat ethnic food, in young adults (18–34 years of age), in those living in larger cities, in those living southern Italy or on the islands, in those with a postgraduate education, in those who are childless, and in those who are employed.

### 3.4. Correlations between the FNS and ODCS Indices

**Figure 1:**
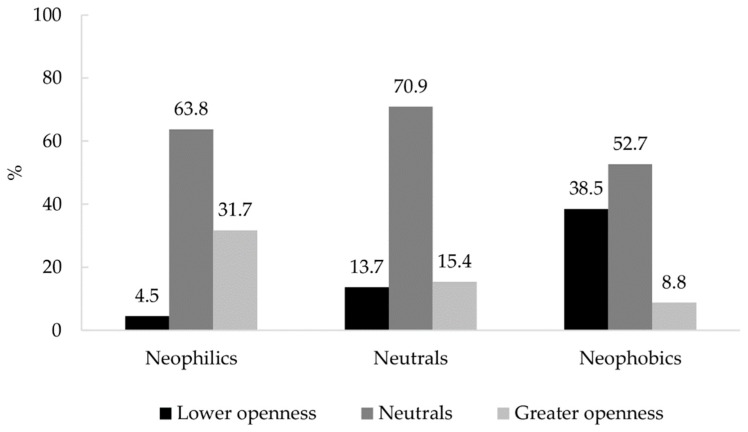
Bivariate analysis of attitudes towards different cultures and of food neophobic groups.

The Pearson correlation coefficient calculated between the FNS and ODCS indices was measured as 0.312 (*p* = 0.000), indicating a positive but weak correlation between the two variables. For the FNS index, the subjects were divided into three groups using one standard deviation from the mean as a cut-off point: Neophilic (17%, score 10–36.4), neutral (69.2%, score 36.5–65.8), and neophobic (13.8%, score 65.9–100). In the same way, three groups were defined using an ODCS index, representing low (15.5%, score 7–25.7), moderate (67.3%, score 25.8–53.5), and high (17.2%, score 53.6–70) levels of openness to different cultures. The bivariate analysis performed on the two variables reveals that among food neophobic subjects, more than four times the number of individuals exhibit less openness than individuals exhibiting more openness. Additionally, only one of seven of those who are neophilic show lower levels of openness rather than higher levels of openness (Figure 1).

## 4. Discussion

The ethnic food market is an important business in Italy. The consumption of ethnic products is widespread [47], and this trend is growing [9,10]. However, ethnic food is far from being part of everyday Italian life.

The FNS and ODCS indices tested with the Italian sample present a high internal reliability coefficient. Our PCA shows that the two scales are not unidimensional and principally load on two factors. The components identified by the FNS separate the regular and reversed items, reflecting the different dimensions measured. These results are consistent with those of previous studies [41,50]. The two components identified by the ODCS recognize the dimensions measured, social interactions, as well as language and mass media use, echoing the findings of Verbeke and Poquiviqui Lopez [39]. 

The Italian sample seems to be slightly neophobic considering that the mean FNS index value is slightly higher than the midpoint of the scale. When we compare this result to the most recent FNS analysis of the Italian context, the samples analyzed by Monteleone [13] and Proserpio [42] seem to be less neophobic than ours and find a midpoint lower than the mean value. Although scales adopting different formats are difficult to compare, several studies suggest that a scale with more response options might produce slightly lower scores [51]. In our study, the 1–10 scale used produced higher scores, which may be attributable to the different sampling and data collection methods applied in the analyzed studies. The data presented in this work were collected from online questionnaires of panels of Italian consumers while in the two studies listed above, participants were recruited on a voluntary basis and completed the questionnaire during experimental sessions.

At the international level, samples showing lower levels of neophobia have been found in other studies [14,16,32,33] while a mean value higher than the midpoint of the scale used has been reported for Lebanese students [50]. A comparison of FNS mean scores of various countries could prove useful in providing an overview of food neophobia levels worldwide. However, generalizations and statistical comparisons are difficult to apply because the scale is sensitive to contexts, as highlighted by previous studies [49].

The mean value of the ODCS index is instead higher than the midpoint value of the scale, suggesting a quite open sample. In analyzing the sociodemographic characteristics, we found that the description of the neophobic consumer appears to differ substantially from that of the consumer with an attitude of openness. The definition of the characteristics of consumers in relation to unfamiliar foods and different cultures deserves special attention for two main reasons. First, it fosters a comprehension of a growing phenomenon. That is, the increasing prevalence of ethnic food not only reflects the proliferation of social change but also has important implications for the food market. Second, the group of neophobic consumers exhibiting less openness to different cultures might have a less varied diet (e.g., Arvola et al. [18] and Schickenberg et al. [23]). The tendency to avoid foods that are different from those usually consumed reduces one’s willingness to try healthy alternatives to known foods [23]. This tendency also reduces one’s willingness to eat certain basic foods, such as vegetable salads and fish [52]. A diet that includes as many different foods as possible is essential to health because it ensures nutritional quality and prevents chemical accumulation due to constant exposure to the same substances [53]. The neophobic trait is recognized as a barrier to diet modification [35] and has a negative influence on diet quality [54] while the willingness to accept different food alternatives can be an indicator of the ability to make positive changes in eating habits.

The results of this study show that men are more neophobic than women. These findings are consistent with other studies [13,32,52]. However, the effect of gender on neophobia remains less clear and varies widely across studies on different regions [31]. According to the data presented here, gender does not influence openness to different cultures. Instead, age seems to play an important role. The results of this study show that levels of food neophobia increase with age, which has already been proven by other studies [13,31,32,33,52]. Openness to different cultures instead declines with age and is characterized as an attitude held by younger age groups. Other studies [52] have highlighted that these results may seem counterintuitive given that with age, an individual should experience more exposure to a broader variety of foods and therefore develop more knowledge of different foods. These factors can promote the acceptance and willingness to try different alternatives. However, such acceptance and willingness could be attributed to a more exploratory and playful attitude even towards food, which can characterize young people, and which can promote curiosity and contact with new foods. Several studies of the Italian context have emphasized that it is precisely young people who adhere to food styles that differ from those of the Mediterranean diet [55] and who are more open to the consumption of ethnic food [56]. These attitudes of young people are probably favored by socialization due to the incremental likelihood of one coming into contact with different cultures relative to the past. In the Italian context, which is characterized by a culinary tradition in which food changes occur slowly, another highly interesting aspect concerns the importance of relationships to the acceptance of a new food. The majority of those reporting consuming ethnic food in Italy reported that they had come into contact with ethnic cuisine through relatives and friends. Italy has a strong gastronomic tradition, and the idea of food is linked to the idea of social relations and sharing, not only among Italians. A study recently conducted in Italy has highlighted how food appears as an element of contact and sharing between natives and migrants. Approximately two out of five immigrants interviewed stated that they have cooked their traditional dishes and have taught their recipes to Italian friends and acquaintances [9]. The social and convivial importance of food and eating practices, which traditionally characterize Italy [2], is recognizable also in Italian consumers’ relationship with ethnic food. Furthermore, the presence of people of different origins and traditions than the Italian ones becomes an opportunity for culinary knowledge and experimentation.

Education, occupation, and income are considered variables that define the socioeconomic status of a person and that may affect opportunities to be exposed to different foods and cultures [27]. The impact of these variables on neophobia has revealed by other studies, whose results are confirmed by the findings of this study: Consumers who are more neophobic are less educated [31,32,52], of lower socioeconomic status [31,52], and, according to the data presented here, are retired. Conversely, consumers exhibiting an attitude of openness towards different cultures are highly educated and employed. Financial status does not emerge as a significant differentiating variable.

In addition, the place of residence (urban vs. rural) was tested as a variable that could affect exposure to new products and to a multicultural environment. This hypothesis has been confirmed by other studies [27,31,32]. However, in this study, being an inhabitant of a large or small city or of a specific area of Italy was not found to affect food neophobia. This result could be related to unique features of the Italian Peninsula that, given its geographic position, is characterized by migratory flows throughout. Otherwise, an openness to different cultures seems to characterize residents of southern Italy and of the islands and of medium-sized cities (100,000–250,000 residents). The relationship between food neophobia, openness to different cultures, and urban living remains an aspect that deserves further study.

The variable “having children” has a significant impact on both food neophobia and openness to different cultures. The most food neophobic individuals declared that they have children while those with more open attitudes were childless. This variable was analyzed because being a parent and the presence of children in the family could result in a more protective attitude and more mistrust of novelty [29,57].

Recent studies have found that the frequency with which ethnic food is consumed is significantly correlated with levels of food neophobia [39,50] and with degrees of openness to other cultures [39]. Our analysis of this aspect in the Italian context presents similar results. Those reporting never having consumed ethnic food in Italy presented a higher FNS index and a lower ODCS index than those declaring that they had consumed such foods. The results of this study confirm the relationship between food neophobia and openness to different cultures as highlighted by other research [14,39].

This study emphasizes a relationship between the psychological trait of neophobia and attitudes towards different cultures, confirming food’s symbolic and cultural value. The studied aspects are immediately evident for ethnic food. These measures have become a crucial indicator of peoples’ levels of willingness to accept elements of novelty in an increasingly globalized and multicultural society. This study analyzed these aspects across a nationwide sample of consumers in Italy. On the one hand, this paper extends the existing literature by providing additional empirical evidence for the Italian context, which has rarely been studied from this point of view. On the other hand, we present an apparently counterintuitive result demonstrating a tendency for young people to be less neophobic and more open to different cultures. This tendency must be studied in more depth in consideration of further social and societal variables. In fact, even if the rooted culinary Italian tradition may seem to be a deterrent to openness to new foods, the very importance attributed to food can serve as an element that encourages sociality, which seemed to emerge from this work.

The acceptance or rejection of a new food involves a complex psychological mechanism that is influenced not only by personal traits but also by specific behaviors and cultural aspects. While the importance of focusing on personality traits and on food neophobia in particular has been demonstrated in several papers, a comprehensive study of these aspects is required to develop a broader account of the phenomenon in Italy as well as to compare it across different contexts. Contact with different cultures through other people, the mass media, travel, and language studies can affect openness to and acceptance of novelty and diversity [40]. The fact that these aspects were not measured in our survey may represent a limitation of the study. Future research should therefore investigate in more depth the actual impact of these aspects in modifying consumers’ attitudes. 

Another limitation of the study is due to the representativeness of the sample. As described in the method section, only gender and geographical area were used in the quota sampling. However the age of the sample was also consistent with that observed in the Italian population over 18 years old. Nevertheless, other sociodemographic variables (i.e., the size of the city of residence, the educational qualification, etc.) that would have made it possible to have a sample more closely related to the Italian population were not considered in the quota sampling,

## 5. Conclusions

This study investigated the relation between food neophobia and openness to different cultures and the consumption of ethnic foods together with sociodemographic variables. The main results show that participants with higher levels of food neophobia do not consume ethnic food while those exhibiting higher levels of openness to different cultures consume it. Moreover, neophobic participants tend to be less open to different cultures. Some sociodemographic variables associated with food neophobia (i.e., gender, age, educational qualifications, the presence of children, occupation, and how well the interviewee’s income meets his or her financial needs) and with an openness to different cultures (i.e., age, location, size of the city of residence, the presence of children, and educational qualifications) were identified. The results of this study may prove useful for those concerned with nutrition as a means to protect public health and who are tasked with designing interventions in nutritional education and communication strategies that address the traits of consumer groups who are reluctant to adopt new proposals. Considering the relation between familiarity with food and a food’s place of origin for its acceptance [52] as well as the importance of cultural and social identification [58], communication activities can be crucial in fostering an awareness of different foods and in promoting a varied and diversified diet.

## Figures and Tables

**Table 1:** Mean values and standard deviations of FNS (Food Neophobia Scale) items and varimax rotated factor loadings.

Item	Mean	SD	Factor 1 ^b^	Factor 2 ^b^
I am constantly sampling new and different food (R) ^a^	5.4	2.6	0.828	−0.046
I don’t trust new foods	6.9	2.3	−0.211	0.770
If I don’t know what is in a food, I won’t try it	5.3	2.8	0.032	0.723
I like foods from different countries (R)	4.9	2.3	0.878	−0.028
Ethnic food looks too weird to eat	6.5	2.4	−0.098	0.752
At dinner parties, I will try a new food (R)	4.4	2.7	0.858	−0.063
I am afraid to eat things I have never had before	6.5	2.5	−0.213	0.794
I am very particular about the foods I will eat	4.3	2.3	0.151	0.472
I will eat almost anything (R)	5.8	2.8	0.703	−0.037
I like to try new ethnic restaurants (R)	5.1	2.7	0.873	−0.086
% variance explained			38	23.3

^a^ R = Reversed items; ^b^ Loadings that are higher on either factor are shown in bold.

**Table 2:** Food neophobia scores by sociodemographic variables.

Variables	N	Mean (± SD)	Range	F	*p*-Value
Gender					
Male	629	51.98 (± 14.37)	14–100	4.246	0.040
Female	688	50.31 (± 15.06)	10–100		
Age				10.070	0.000
18–34	297	48.51 (± 15.27)	10–98		
35–54	487	50.50 (± 14.94)	14–100		
>55	533	53.11 (± 14.02)	14–98		
Location				1.098	0.349
Northwestern Italy	346	50.07 (± 15.03)	10–100		
Northeastern Italy	252	52.15 (± 15.71)	10–100		
Central Italy	256	51.62 (± 16.38)	14–98		
Southern Italy and islands ^a^	463	51.02 (± 12.94)	14–94		
Size of city of residence				2.548	0.054
<30,000	512	52.36 (± 15.24)	10–100		
30,001–100,000	365	50.46 (± 13.91)	15–98		
100,001–250,000	160	51.32 (± 14.35)	10–93		
>250,000	280	49.54 (± 15.01)	14–100		
Educational qualifications				4.679	0.000
Elementary/lower	130	54.14 (± 14.36)	14–98		
secondary school diploma					
Vocational qualification	70	52.93 (± 15.12)	22–92		
Upper secondary school	607	51.94 (± 14.29)	14–100		
diploma					
Higher education diploma	52	53.48 (± 12.99)	24–85		
Degree	387	48.61 (± 15.16)	10–100		
Postgraduate qualification	72	48.52 (± 15.94)	20–100		
Children				7.451	0.006
Yes	804	51.99 (± 14.38)	14–100		
No	513	49.72 (± 15.23)	10–100		
Occupation				11.129	0.000
Student/looking for first job	155	48.99 (± 14.48)	14–98		
Homemaker	154	53.20 (± 13.48)	18–91		
Retired	244	55.37 (± 13.47)	14–98		
Unemployed	138	53.36 (± 13.89)	19–94		
Employed	626	48.95 (± 15.31)	10–100		
Meeting				8.336	0.000
financial responsibilities					
Very easy	121	46.94 (± 16.06)	10–100		
Quite easy	498	50.96 (± 14.33)	14–100		
With some difficulty	545	50.87 (± 14.91)	14–100		
With much difficulty	153	55.70 (± 13.64)	14–100		
Ethnic food consumption				141.289	0.000
Yes	1116	49.15 (± 14.26)	10–100		
No	201	61.92 (± 12.63)	19–100		

^a^ Islands include Sicily and Sardinia.

**Table 3:** Mean values and standard deviations of ODCS (Openess to Different Cultures Scale) items and varimax rotated factor loadings.

Item	Mean	SD	Factor 1 ^a^	Factor 2 ^a^
1. I am very comfortable dealing with non-Italians	6.6	2.2	0.879	0.184
2. I like to go to places where I can be among non-Italians	6.1	2.4	0.881	0.261
3. I like to participate in activities of non-Italians	5.9	2.3	0.883	0.295
4. Some of my friends are non-Italians	6.3	2.7	0.632	0.353
5. I often read foreign newspapers or magazines	4.4	2.8	0.293	0.850
6. I often watch foreign television	4.4	2.9	0.223	0.890
7. I like to study foreign languages	5.9	2.7	0.258	0.749
% variance explained			60	16

^a^ Loadings that are higher on either factor are shown in bold.

**Table 4:** Attitudes towards different cultures based on sociodemographic variables.

Variables	N	Mean (± SD)	Range	F	*p*-Value
Gender				3.644	0.056
Male	629	38.80 (± 13.70)	7–70		
Female	688	40.26 (± 13.99)	7–70		
Age				13.610	0.000
18–34	297	41.83 (± 13.86)	7–70		
35–54	487	40.75 (± 13.92)	7–70		
>55	533	37.22 (± 13.49)	7–70		
Location				7.649	0.000
Northwestern Italy	346	38.65 (± 13.30)	7–70		
Northeastern Italy	252	37.72 (± 13.72)	7–70		
Central Italy	256	38.23 (± 14.47)	7–70		
Southern Italy and islands ^a^	463	42.00 (± 13.72)	7–70		
Size of city of residence				4.851	0.002
<30,000	512	37.80 (± 13.30)	7–70		
30,001–100,000	365	40.26 (± 13.31)	7–70		
100,001–250,000	160	41.37 (± 16.24)	7–70		
>250,000	280	40.87 (± 13.84)	7–70		
Educational qualifications				12.253	0.000
Elementary/lower secondary school diploma	130	35.48 (± 13.98)	7–66		
Vocational qualification	70	35.47 (± 14.56)	7–70		
Upper secondary school diploma	607	38.23 (± 13.18)	7–70		
Higher education diploma	52	38.32 (± 13.17)	10–70		
Degree	387	42.78 (± 13.56)	7–70		
Postgraduate qualification	72	45.81 (± 15.41)	7–70		
Children				4.498	0.034
Yes	804	38.92 (± 14.09)	7–70		
No	513	40.58 (± 13.45)	7–70		
Occupation				8.840	0.000
Student/looking for first job	155	40.50 (± 12.47)	7–70		
Homemaker	154	37.05 (± 13.78)	7–70		
Retired	244	35.78 (± 13.12)	7–66		
Unemployed	138	39.87 (± 13.57)	9–70		
Employed	626	41.36 (± 14.22)	7–70		
Meeting financial responsibilities				2.480	0.060
Very easy	121	40.71 (± 13.92)	7–70		
Quite easy	498	39.79 (± 13.18)	7–70		
With some difficulty	545	39.89 (± 14.31)	7–70		
With much difficulty	153	36.78 (± 14.21)	7–70		
Ethnic food consumption				70.514	0.000
Yes	1116	40.89 (± 13.59)	7–70		
No	201	32.21 (± 13.03)	7–70		

^a^ Islands include Sicily and Sardinia.
